# Mapping an index of the myelin *g*-ratio in infants using magnetic resonance imaging

**DOI:** 10.1016/j.neuroimage.2016.02.040

**Published:** 2016-05-15

**Authors:** Douglas C. Dean, Jonathan O'Muircheartaigh, Holly Dirks, Brittany G. Travers, Nagesh Adluru, Andrew L. Alexander, Sean C.L. Deoni

**Affiliations:** a Waisman Center, University of Wisconsin-Madison, Madison, WI 53705, USA; b Department of Neuroimaging, King's College London, Institute of Psychiatry, London SE5 8AF, UK; c Department of Kinesiology, University of Wisconsin-Madison, Madison, WI 53705, USA; d Advanced Baby Imaging Lab, Brown University School of Engineering, Providence, RI 02912, USA; e Department of Psychiatry, University of Wisconsin-Madison, Madison, WI 53719, USA; f Department of Medical Physics, University of Wisconsin-Madison, Madison, WI 53705, USA; g Department of Pediatric Radiology, Children's Hospital Colorado, Aurora, CO, USA; h Department of Radiology, University of Colorado Denver, Denver, CO, USA

## Abstract

Optimal myelination of neuronal axons is essential for effective brain and cognitive function. The ratio of the axon diameter to the outer fiber diameter, known as the *g*-ratio, is a reliable measure to assess axonal myelination and is an important index reflecting the efficiency and maximal conduction velocity of white matter pathways. Although advanced neuroimaging techniques including multicomponent relaxometry (MCR) and diffusion tensor imaging afford insight into the microstructural characteristics of brain tissue, by themselves they do not allow direct analysis of the myelin *g*-ratio. Here, we show that by combining myelin content information (obtained with mcDESPOT MCR) with neurite density information (obtained through NODDI diffusion imaging) an index of the myelin *g*-ratio may be estimated. Using this framework, we present the first quantitative study of myelin *g*-ratio index changes across childhood, examining 18 typically developing children 3 months to 7.5 years of age. We report a spatio-temporal pattern of maturation that is consistent with histological and developmental MRI studies, as well as theoretical studies of the myelin *g*-ratio. This work represents the first ever *in vivo* visualization of the evolution of white matter *g-*ratio indices throughout early childhood.

## Highlights

• Multi-modal MRI method that combines mcDESPOT and NODDI to form an index of myelin *g*-ratio.
• Myelin *g*-ratio index measured in typically developing children, for the first time
• Developmental trajectories of NODDI and myelin *g*-ratio index across white matter
• Prospect of myelin *g*-ratio index for neurodevelopmental disorders is described.

## Introduction

The maturation of the brain's white matter and the establishment of the lipid myelin sheath around neuronal axons are critical processes in human brain development. Beginning prenatally, myelination advances rapidly over the first 2 years of life before slowing through childhood and continuing to slowly develop into the second and third decades of life (Barkovich et al., 1988, Bartzokis et al., 2010, Brody et al., 1987, Paus et al., 2001). The spatiotemporal pattern of myelination follows a carefully choreographed order, extending from deep to superficial brain regions in a posterior to anterior pattern (Barkovich et al., 1988, Brody et al., 1987, Paus et al., 2001). This temporal pattern coincides with the emergence and refinement of cognitive and behavioral functions (Fields, 2008, Nagy et al., 2004), with the neural activity itself partly driving myelination by oligodendricytes (Fields, 2005, Ishibashi et al., 2006, Ullén, 2009).

The primary role of the myelin sheath is to increase the conduction velocity of electrical impulses along the myelinated fiber. For a fixed axon diameter, conduction velocity increases in proportion to myelin thickness (Waxman, 1980). However, biophysical limitations, including intracranial space volume, axonal energy consumption; and other neurodevelopmental processes (dendritic arborization, synapse formation, and neuronal pruning) influence the degree of myelination (Chomiak and Hu, 2009). As a result, it is hypothesized that an optimal ratio exists between the axon diameter and the total fiber diameter (which consists of both the axon diameter and the thickness of the myelin sheath) that maximizes transduction efficiency (Chomiak and Hu, 2009, Goldman and Albus, 1968, Rushton, 1951). Defined as the *g*-ratio, this index is informative of the underlying myelin microstructure, and may indicate the relative efficiency and maximal conduction velocity of particular axons and white matter pathways.

Rushton (1951) first proposed that the relationship between axonal conduction velocity was interdependent on fiber diameter and myelin thickness. Through a simple mathematical model, he suggested that a *g*-ratio of 0.6 would yield optimum fiber conduction. This result was found to agree well with experimental measurements in peripheral nerve fibers (Hursh, 1939). However, central nervous system (CNS) fibers are generally smaller, and *g*-ratios greater than 0.6 are commonly observed (Goldman and Albus, 1968). To account for this discrepancy, Chomiak and Hu revisited Rushton's initial analysis and theoretical framework and included additional biophysical constraints within their model of impulse conduction. This work suggested an optimal *g*-ratio of 0.77 for CNS fibers, which agree more closely with experimentally measured *g*-ratios in the rat brain (Chomiak and Hu, 2009).

Outside of these theoretical models, however, there exists significant variation in the ratio of myelin thickness to fiber diameter between different brain regions, in different regions along the same axons, and across neurodevelopment (Graf von Keyserlingk and Schramm, 1984, Ikeda and Oka, 2012, Sanders and Whitteridge, 1946, Sherman and Brophy, 2005, Spencer et al., 1973). Although not specifically tested, neurodevelopmental differences between males and females have been hypothesized to be associated with variations in the myelin *g*-ratio (Paus and Toro, 2009, Pesaresi et al., 2015), and individuals with schizophrenia are also believed to have atypical *g-*ratio values (Du and Ongür, 2013).

Despite the importance of myelin thickness to neuronal communication and consequently normative brain function, *in vivo* measurement of this parameter has remained limited. Recently, several mathematical models have been proposed that relate MRI-derived measures of myelin content (obtained using magnetization transfer or multicomponent relaxometry imaging) and fiber volume fraction (obtained from diffusion weighted imaging) to the myelin *g*-ratio. Stikov et al., for example, proposed a tissue model that utilizes measurements of the bound pool fraction (F) from quantitative magnetization transfer imaging (qMT) with measurements of fractional anisotropy from diffusion tensor imaging (DTI) to estimate an aggregate measure of the myelin *g-*ratio (Stikov et al., 2011). Building on this result, Campbell et al. examined the differences in the tissue model when using DTI based parameters *versus* the neurite orientation dispersion and density imaging (NODDI) model parameters (H. Zhang et al., 2012), and found that NODDI-based measures were more robust for estimating the fiber volume fraction compared to DTI measures (Campbell et al., 2014). Stikov et al. further showed MRI derived *g-*ratio estimates are consistent with histological estimates in the corpus callosum of five macque monkeys (Stikov et al., 2015a, Stikov et al., 2015b), and have demonstrated whole-brain *g-*ratio mapping may be an informative biomarker in multiple sclerosis (Stikov et al., 2015a). Using a similar theoretical framework, Mohammadi et al. additionally demonstrated histologically consistent *in vivo* MR measurements of *g*-ratio in 37 healthy volunteers across the whole brain (Mohammadi et al., 2015). These studies provide initial evidence that suggests it is possible to use MRI to map an aggregate measure of the myelin *g*-ratio *in vivo* and that such measurements are histologically consistent. These studies have additionally used qMT measurements, which have been shown to correlate with histological estimates of myelin (Schmierer et al., 2007, Schmierer et al., 2008, Thiessen et al., 2013), to derive the myelin volume fraction component of the *g-*ratio formulation. Recent research has also suggested that alternative quantitative imaging techniques, such as multicomponent relaxometry, may be sensitive to the underlying myelin content (Alexander et al., 2011, Deoni, 2010). Thus these studies raise the possibility that such alternative techniques may additionally be appropriate for *in vivo* estimation of a myelin *g*-ratio index.

Multicomponent relaxometry (MCR) estimates specific MR tissue characteristics by decomposing the measured MRI signal into contributions from distinct microstructural water pools with different T_1_ and T_2_ relaxation times. Prior MCR studies have consistently reported at least two water compartments: a fast-relaxing water pool attributed to water trapped between the myelin-lipid bilayers; and a slower-relaxing water pool attributed to intra-/extra-cellular water (MacKay et al., 1994, MacKay et al., 2006). Quantification of the signal from the myelin-bound water, termed the myelin water fraction (MWF), has been shown to strongly correlate with histological assessments of myelin content (Laule et al., 2006, Laule et al., 2008), thus warrants the use of such a parameter for assessing myelin content (Laule et al., 2008). While MCR has traditionally been performed using multiple spin-echo T_2_ decay data (Whittall et al., 1997), a recent approach named mcDESPOT (multi-component driven equilibrium single pulse observation of T1 and T2), has been proposed, which utilizes variable flip angle measurements from steady state pulse sequences (Deoni et al., 2013b, Deoni et al., 2008) Using mcDESPOT, our group has previously demonstrated a spatio-temporal pattern of myelination throughout infant neurodevelopment that closely mirrors the established histological time-line (Dean et al., 2014b, Deoni et al., 2011, Deoni et al., 2012). However, it remains unclear how the myelin *g*-ratio changes with age throughout development and in association with behavioral maturation.

In this work, we present the first report of indices of the myelin *g*-ratio across neurodevelopment using magnetic resonance imaging (MRI). We extend our prior analyses of early neurodevelopment by combining mcDESPOT myelin water volume fraction (VF_M_) and NODDI fiber volume fraction data to obtain whole-brain voxel-wise maps of an apparent myelin *g*-ratio index. Further, we sought to explore the evolution of this *g*-ratio index across early neurodevelopment by tracking these measures in 18 healthy infants and toddlers between 3 months and 7.5 years of age. Within white matter, we demonstrate *g*-ratio index *versus* age trajectory that asymptotically approaches 0.8, consistent with theoretical predictions of the myelin *g*-ratio.

## Materials and methods

### Subjects

Participants in this study were a subset of those involved in a broader longitudinal investigation of white matter maturation in healthy, typically developing children and corresponding behavioral development (Dean et al., 2014b, Deoni et al., 2012). Informed parental consent was obtained in accordance to ethics approval from the Institutional Review Board of Brown University. Children enrolled in the study met the following inclusion/exclusion criteria: uncomplicated single birth between 37 and 42 weeks; no *in utero* exposure to alcohol or illicit drugs; no familial history of major psychiatric or depressive illness; no diagnosis of major psychiatric, depressive or learning disorder in participant; and no pre-existing neurological conditions or major head trauma in participant. A total of 18 healthy infants (13 males/5 females) and toddlers between 102 and 2713 days of age (approximately 3 months to 7.5 years, mean = 2.58 years), corrected for a 40-week gestation were included in the present study. The NODDI imaging protocol was added late into the longitudinal study and thus these 18 study participants were the only subjects that had both mcDESPOT and NODDI data. Full participant demographics are provided in Table 1.

**Table 1:** Demographic information for the 18 study participants.

Subject	Sex	Age (days)	gestational period (weeks)	Birth weight (kg)	Birth height (cm)
1	F	102	38.57	2.78	47.63
2	M	116	39.57	2.72	45.72
3	F	123	40.71	4.00	54.61
4	M	124	40.86	2.89	45.72
5	M	129	40.71	NR	NR
6	M	354	38.29	2.75	48.26
7	M	357	38.00	2.78	49.53
8	M	362	39.00	3.20	50.80
9	M	376	41.00	3.20	48.26
10	M	663	38.00	2.86	48.26
11	M	717	39.00	3.83	53.34
12	M	940	39.86	2.89	50.80
13	M	1228	39.43	3.74	54.99
14	M	1381	39.00	3.43	50.80
15	M	1978	40.57	2.98	53.34
16	F	2334	39.86	2.98	59.17
17	F	2713	38.00	4.03	52.07
18	F	2713	42.00	3.12	NR

NR = not reported.

### Data acquisition

Children under 4 years of age were scanned during natural, non-sedated, sleep; while children over this age were able to watch a favorite TV show or movie while being scanned (Dean et al., 2014c). All data was acquired on a 3 Tesla Siemens Tim Trio scanner equipped with a 12-channel receive-only head RF array coil. To minimize intra-scan motion, children were swaddled with an infant or pediatric MedVac vacuum immobilization bag (CFI Medical Solutions, USA) and foam cushions were placed around their head. Scanner noise was reduced by limiting the peak gradient amplitudes and slew-rates to 25 mT/m/s. A noise-insulating insert (Quiet Barrier HD Composite, UltraBarrier, USA) was also fitted to the inside of the scanner bore. MiniMuff pediatric ear covers and electrodynamic headphones (MR Confon, Germany) were used for all scanned children. A pediatric pulse-oximetry system and infrared camera were used to continuously monitor the infants and children during scanning.

#### mcDESPOT imaging

Age-specific and acoustically muffled imaging protocols, previously described in Deoni et al. (2012), were used to acquire mcDESPOT imaging data from each subject. Each mcDESPOT protocol consisted of 8 T_1_-weighted spoiled gradient echo images (SPGR or spoiled FLASH) and 16 balanced T_1_/T_2_-weighted steady-state free precession (bSSFP or TrueFISP) images acquired across multiple flip angles (Deoni et al., 2013b). Two inversion-prepared (IR)-SPGR images were additionally acquired for correction of radio-frequency (B_1_) inhomogeneities and bSSFP images were acquired with two phase cycling patterns (φ = 180^o^ and 0^o^) for correction of main magnetic field (B_0_) inhomogeneities (Deoni, 2011). Since the field-of-view and image-matrix size varied between each of the age-specific imaging protocols that were designed (to account for the variable head size of the subject population of the longitudinal study while maintaining a constant 1.8 mm isotropic voxel resolution (Deoni et al., 2012)) total imaging times ranged from 19 min for the youngest infants, to 24 min for older and larger children.

#### NODDI imaging

A two-shell diffusion weighted imaging (DWI) protocol was additionally acquired using a single-shot, spin-echo, echo planar imaging (EPI) pulse sequence. In order to make the acoustic noise of this scan tolerable for natural sleep, the sequence was additionally modified by reducing the gradient switching rate and maximum gradient amplitude to 60% and 80% of maximum, respectively. Diffusion weighting was performed with bipolar gradients with dual-echo refocusing to reduce eddy currents (Reese et al., 2003). Parallel acquisition, with a geometric reduction factor of two, was used to reduce image distortions from magnetic field inhomogeneities and reduce acquisition time. Diffusion-weighted images were obtained in thirty non-collinear diffusion encoding directions with *b* = 700 and 2000 s/mm^2^, and two *b* = 0 images. Forty-eight contiguous 2.5 mm axial slices were acquired over the cerebrum and cerebellum (matrix = 88 × 88; Field of view [FOV] = 220 mm; resolution = 2.5 × 2.5 × 2.5 mm^3^; repetition time [TR] = 6700 ms; echo time [TE] = 104 ms and pixel bandwidth = 1623 Hz). Total imaging time was 9 min 49 s.

### Image analysis and *g*-ratio index calculation

Following acquisition, data were assessed for motion artifacts (blurring, ghosting, etc) and standard mcDESPOT processing was performed (Deoni et al., 2012). Individual SPGR, IR-SPGR, and bSSFP images for each participant were linearly co-registered to account for subtle head movement during the scan (Jenkinson et al., 2002) and non-parenchyma voxels were removed using an automated and deformable model approach (S. M. Smith, 2002). Corrections for flip angle errors and off-resonance inhomogeneities were calculated using the DESPOT1-HIFI and DESPOT2-FM techniques, respectively (Deoni, 2011). The SPGR and bSSFP data were subsequently fit to a 3-pool tissue model that estimates the volume fractions and relaxation times for intra/extra-axonal water, myelin-associated water, and non-exchanging free water. The volume fraction associated with the myelin water compartment of the model therefore provides VF_M_ estimates at each image voxel (Deoni et al., 2013a, Deoni et al., 2013b).

NODDI images were corrected for distortion, translation and rotation from eddy currents and bulk head motion using an affine registration tools implemented in the fMRIB Diffusion Toolbox, and the gradient orientations were corrected for rotation. The pre-processed data were then fit to a three-compartment tissue model to provide neurite density and dispersion estimates (H. Zhang et al., 2012) using an available MATLAB toolbox (nitrc.org) and adapting it to run on condor parallel computing environment (https://github.com/nadluru/NeuroImgMatlabCondor). Default model assumptions and fixed parameter values as described in Zhang et al. (2012) were used in the fitting of the NODDI model. From this model fit, the tissue model parameters corresponding to the volume fraction of the intra-cellular or restricted diffusion compartment (ν_IC_) and the volume fraction of an isotropic diffusion compartment (ν_ISO_) are estimated. Within the NODDI formulation, restricted diffusion is attributed to axons and dendrites (neurites) and thus ν_ic_ is interpreted as a quantitative measure of neurite density, while the volume fraction of the isotropic diffusion compartment, ν_iso_, is attributed to CSF or isotropic diffusion (H. Zhang et al., 2012).

### Myelin *g*-ratio index calculation

To calculate the myelin *g*-ratio index, each participant's mcDESPOT and NODDI parameter maps were first co-registered as follows: for each infant, a mean non-diffusion weighted image was calculated from the two acquired b = 0 images. This image was then registered to the infant's high flip angle T_1_-weighted SPGR image using an automatic affine registration technique (Jenkinson et al., 2002). The calculated transformation matrix was then applied to the restricted (ν_IC_) and isotropic (ν_ISO_) volume fraction maps estimated from the NODDI data. Finally, the *g*-ratio index was calculated from the VF_M_, ν_IC_, and ν_ISO_ maps as in Stikov et al. (2015a),

(1) $${\mathrm{VF}}_{A}=\left(1-{\mathrm{VF}}_{M}\right)\left(1-\nu_{\mathrm{ISO}}\right)\nu_{\mathrm{IC}}$$

(2) $${\mathrm{VF}}_{F}={\mathrm{VF}}_{M}+{\mathrm{VF}}_{A}$$

and

(3) $$g=\sqrt{1-\frac{{\mathrm{VF}}_{M}}{{\mathrm{VF}}_{F}}}$$

Here, VF_A_ denotes the axon volume fraction, and VF_F_ is the total fiber (sum of the myelin and axon) volume fraction.

### Reconstruction of *g*-ratio developmental trajectories

In order to generate the developmental trajectories of regional myelin g-ratio indices, the image data for all the subjects were spatially normalized using nonlinear diffeomorphic image registration (Avants et al., 2008) and the subjects high flip angle SPGR T_1_-weighted image to transform between each infant's image space and a previously created study-specific template (Deoni et al., 2012). An adult reference brain template was also non-linearly registered to the infant template to provide brain region tissue masks, as described in Deoni et al. (2012). Anatomical regions of interest (Mazziotta et al., 2001, Oishi et al., 2008) within this co-registered reference dataset, including left and right hemisphere frontal, occipital, parietal, temporal, and cerebellar white matter; genu, body, and splenium of the corpus callosum; left and right hemisphere cingulum, internal capsules, corona radiata, and optic radiations, were then superimposed onto each infant's VF_M_, VF_F_, ν_IC_, ν_ISO_, and myelin *g*-ratio index maps. Mean and standard deviation values for each parameter were calculated for each region and plotted with respect to age.

Pearson partial correlations between computed myelin *g-*ratio index and VF_M_; myelin *g-*ratio index and VF_F_; myelin *g-*ratio index and ν_IC_; and myelin *g*-ratio index and ν_ISO_ were calculated for each white matter tract and region while taking into account the age of subjects.

#### Modeling *g*-ratio index trajectories

To characterize developmental trajectories, logarithmic curve models of the form *g-ratio*(age) = αln(age) − β, were fit to the mean myelin *g*-ratio index data for each brain region and white matter tract. Potential hemispheric differences were tested by fitting the data to each hemisphere independently (dual-curve model) as well as average combined (single-curve model). An F-test was used to determine whether the dual-curve model was justified and to identify areas with hemispheric maturation rate differences.

## Results

**Fig. 1:**
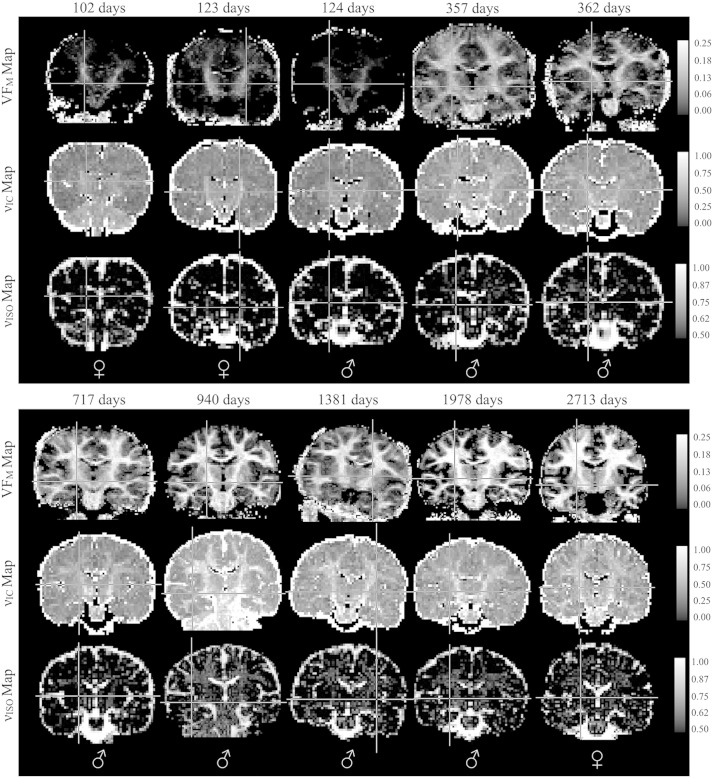
Representative raw coronal mcDESPOT VF_M_ and NODDI ν_IC_ and ν_ISO_ parameter maps.

**Fig. 2:**
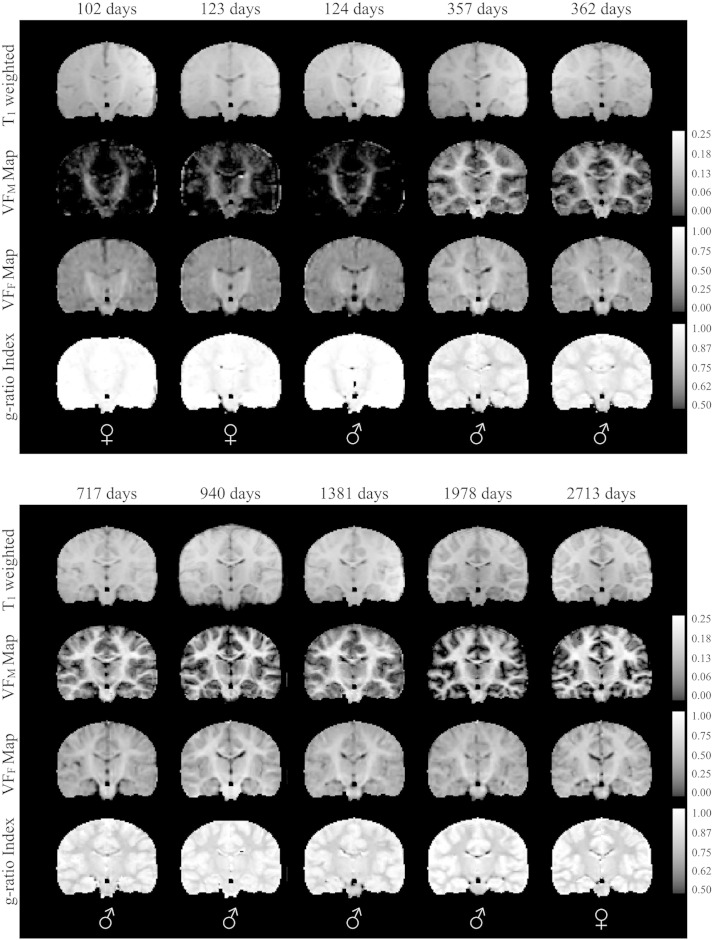
Normalized coronal T_1_-weighted and quantitative VF_M_, VF_F_, and myelin *g*-ratio index maps for representative subset of typically developing subjects. Images are shown in radiological convention (viewing left = anatomical right). The sex of each infant is denoted in the bottom panel. Note: while *g-*ratio in gray matter has limited interpretation, these areas have not been masked in the *g*-ratio index maps so that changes in myelination can be better appreciated.

**Fig. 3:**
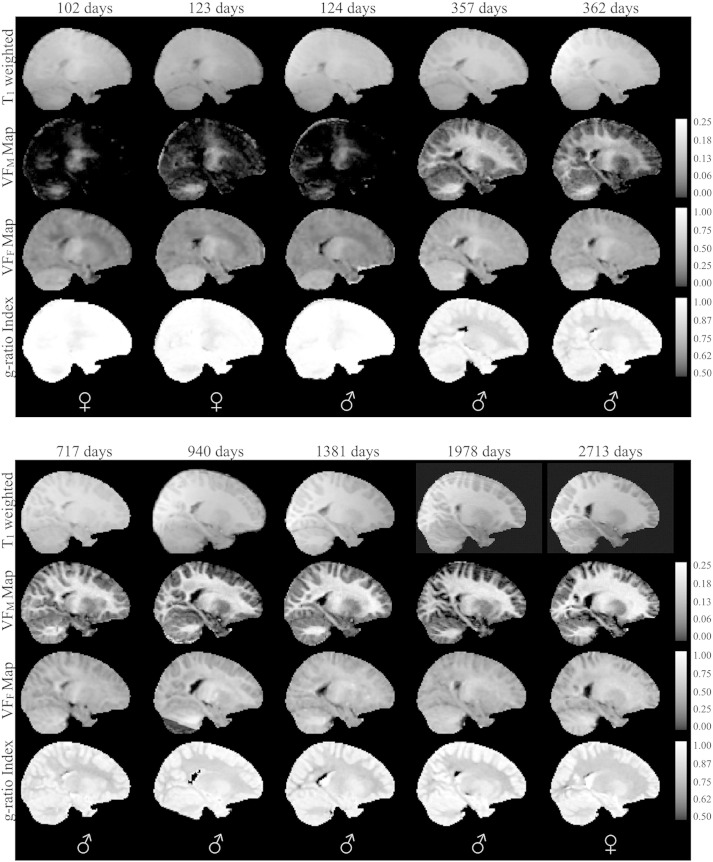
Normalized sagittal T_1_-weighted and quantitative VF_M_, VF_F_, and myelin *g*-ratio index maps for representative subset of the typically developing subjects. The sex of each infant is denoted in the bottom panel. Right hemisphere is shown. Note: while *g-*ratio in gray matter has limited interpretation, these areas have not been masked in the *g*-ratio index maps so that changes in myelination can be better appreciated.

Representative coronal images of raw mcDESPOT derived VF_M_ maps and NODDI ν_IC_ and ν_ISO_ parameter maps from 10 representative subjects are shown in Fig. 1, while reformatted coronal and sagittal images from the spatially normalized whole-brain T_1_ weighted, and quantitative VF_M_, VF_F_, and *g*-ratio index maps of the same 10 infants are shown in Figs 2 and 3, respectively. The expected shift in the gray/white matter contrast across the T_1_-weighted images is evident, reflecting the changes in the underlying tissue content that have been described to take place during this developmental stage (Barkovich et al., 1988, Dietrich et al., 1988, Paus et al., 2001). Myelin maturation throughout the brain is further reflected through the spatio-temporal patterns of the quantitative VF_M_, VF_F_, and myelin *g*-ratio index measurements. These maps detail a specific myelination pattern, beginning in the cerebellum and internal capsules, advancing to the splenium of the corpus callosum and optic radiations, and finally extending to the occipital, parietal, temporal and frontal lobes. This general center-out, posterior–anterior pattern mirrors the general myelination pattern that has been well documented from histological studies (Barkovich et al., 1988, Kinney et al., 1988, Sidman and Rakic, 1982).

**Fig. 4:**
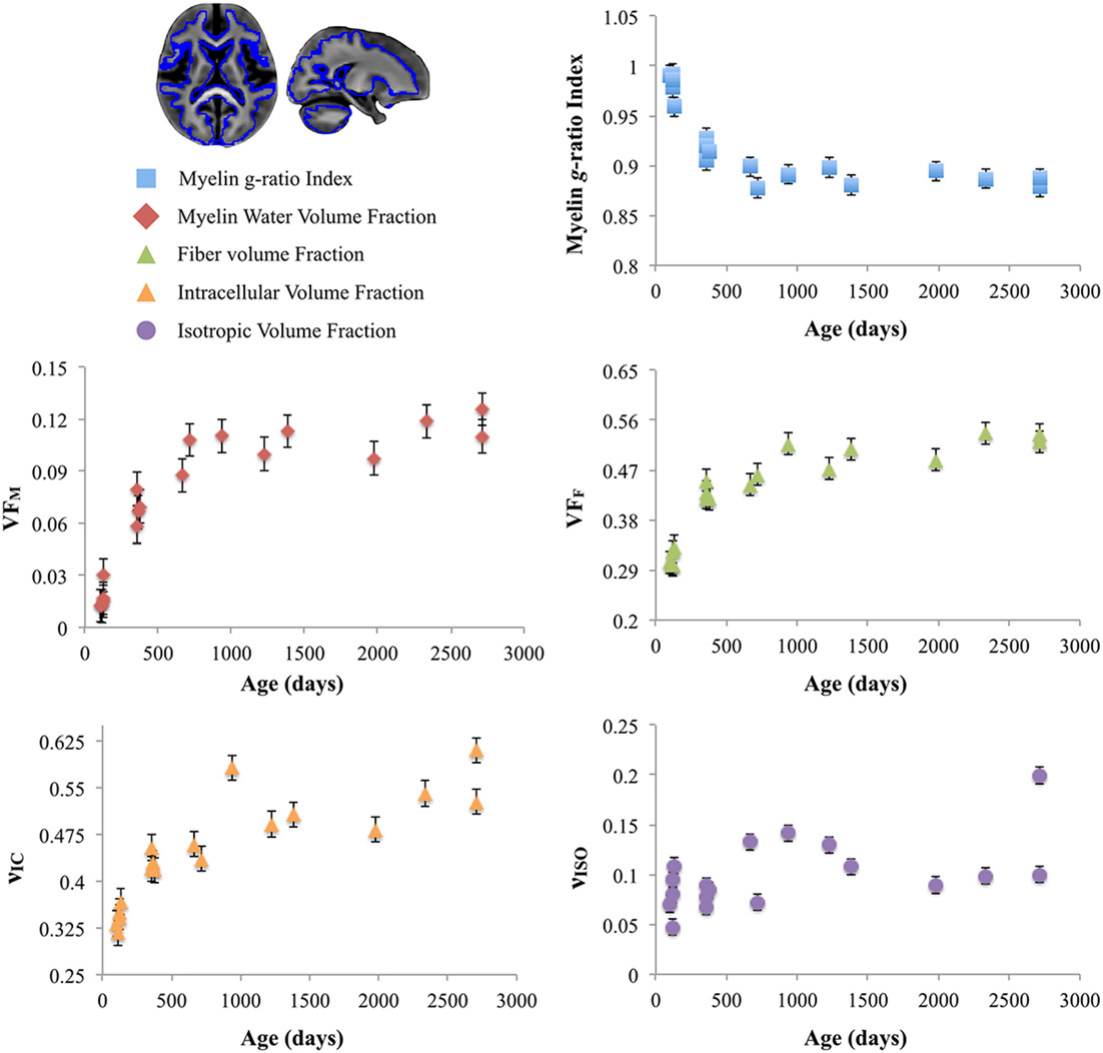
Representative trajectories of the myelin g-ratio index (blue), VF_M_ (red), VF_F_ (green), *ν*_*ic*_ (orange), and *ν*_*iso*_ (purple) for the mean white matter, outlined in blue on the study-specific template. error bars represent the standard deviation of the measurement. Myelin *g*-ratio index trajectories reveal a decreasing logarithmic trajectory that approaches optimal theoretical estimates of 0.8.

Quantitative trajectories of mean white matter myelin *g*-ratio index, VF_M_, VF_F_, ν_IC_, and ν_ISO_ are shown in Fig. 4. These developmental trajectories reveal predominantly nonlinear growth pattern, with VF_F_ and ν_IC_ increasing logarithmically with age and VF_M_ following an approximate sigmoidal pattern (demonstrated previously in Dean et al., 2014b, Dean et al., 2014a). *g*-Ratio index estimates in white matter decreased logarithmically and asymptotically approached the theoretical optimal estimates of 0.8 (Chomiak and Hu, 2009). The differences in the shape of these trajectories indicate the differential sensitivity of these measures to the structural characteristics of white matter. While little myelin is present at birth, reflected by the near-zero VF_M_ and large *g*-ratio index values at the beginning of the trajectory, the structural foundation for myelin (neurons/axons) exists. The presence of this microstructure gives rise to the non-zero measures of VF_F_ and ν_IC_ throughout the entire age-range.

**Fig. 5:**
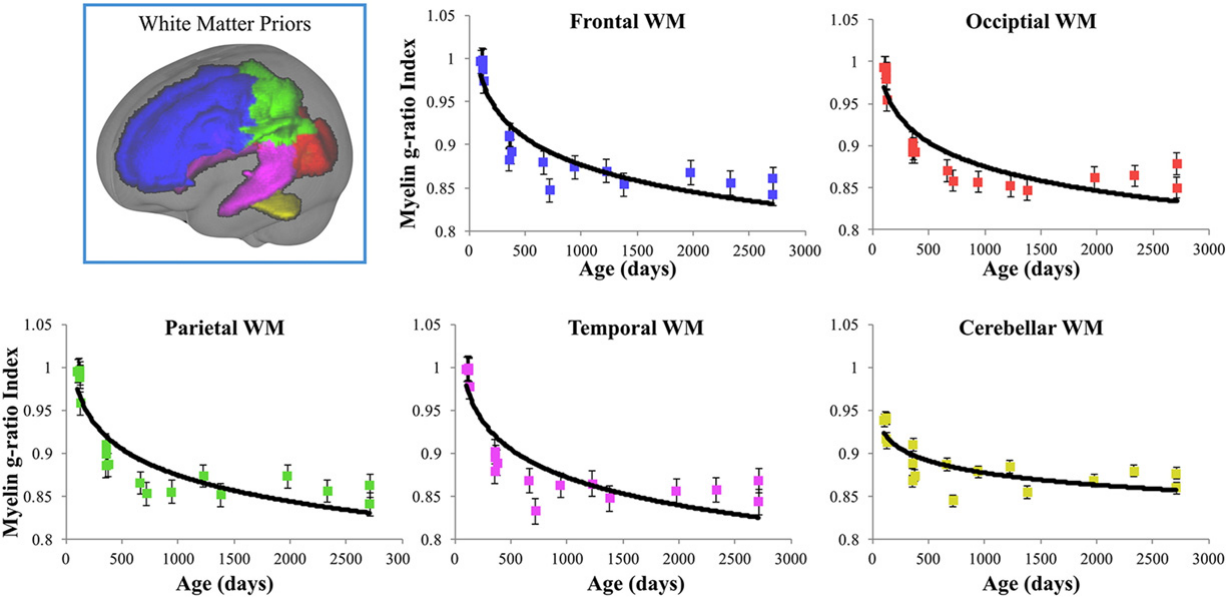
Myelin *g*-ratio index trajectories and corresponding logarithmic fits for frontal, occipital parietal, temporal, and cerebellar white matter.

**Fig. 6:**
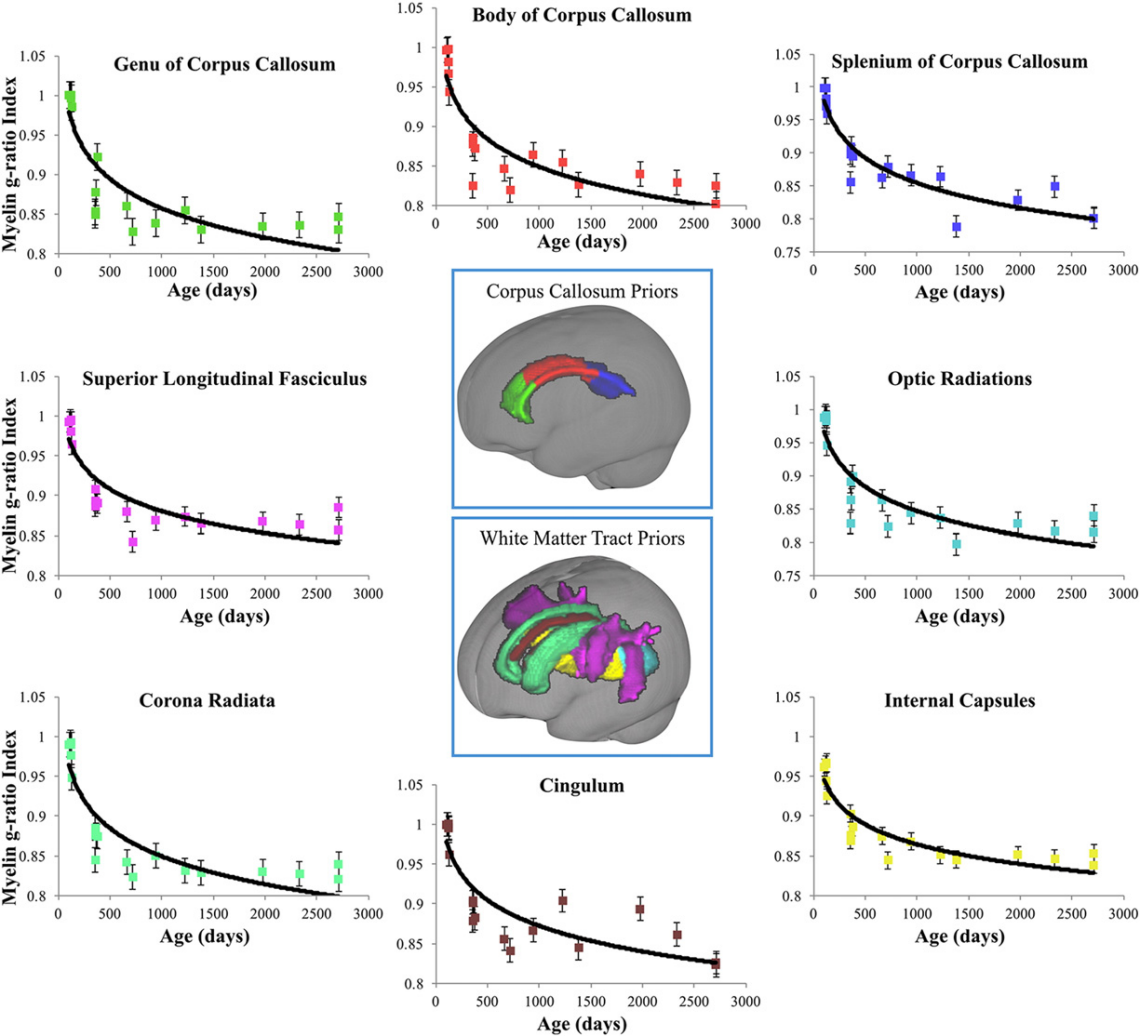
Myelin *g*-ratio index trajectories and corresponding logarithmic fits for genu, body, and splenium of the corpus callosum; cingulum, corona radiata, optic radiations, internal capsules, and superior longitudinal fasciculus.

**Fig. 7:**
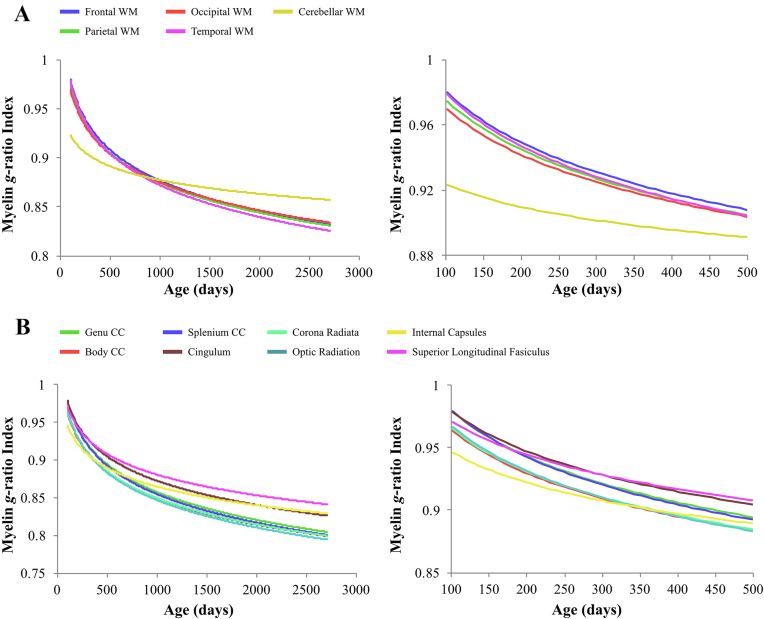
Comparison of reconstructed myelin *g*-ratio index trajectories for different white matter regions (A); corpus callosum and white matter tracts (B). The left panel represents the trajectory of myelin g-ratio index across the full age range, while the right panel displays the first 500 days of development.

To examine the evolution of the myelin *g-*ratio index with age, logarithmic functions were fit to the mean *g*-ratio index *versus* age data for frontal, occipital, parietal, temporal, and cerebellar white matter, as well as the body, splenium and genu of the corpus callosum, and the internal capsules, cingulum, corona radiata, optic radiations, and superior longitudinal fasciculus. For all bilateral regions, functions were fit to right and left hemisphere data independently. Regional mean trajectories and model fits are displayed in Figs. 5 and 6 for these regions, and a comparison of trajectories across all regions is shown in Fig. 7. A summary of the logarithmic curve equations calculated for each region is shown in Table 2. While all regions were found to follow the same logarithmically decreasing pattern, we identified regional variation in the onset and rate of myelination. For example, cerebellar white matter is observed to mature prior to other white matter regions, with myelin present at birth, and frontal white matter having the slowest rate of myelin development. This pattern mirrors prior histological studies (Barkovich et al., 1988, Kinney et al., 1988, Sidman and Rakic, 1982). Using the derived regional *g*-ratio index growth trajectories (Table 2), we extrapolated to 10,000 days to determine the approximate asymptotic value. These values are shown in Table 3 and, with an average value of 0.78 (range 0.71 to 0.9), agree well with the predicted value of 0.8 (Chomiak and Hu, 2009).

**Table 2:** Summary of calculated myelin *g*-ratio index logarithmic fits to each hemispheric brain region. An F-test was used to determine if the data justified modeling the data independently. Values in bold type denote regions where the right and left hemisphere data were significantly different (p < 0.05 uncorrected).

Region/tract	Left hemispheric equation	Right hemispheric equation	F stat
Frontal WM	− 0.046 ∗ ln(Age) + 1.1936	− 0.044 ∗ ln(Age) + 1.1837	**0.0049**
Occipital WM	− 0.044 ∗ ln(Age) + 1.1753	− 0.038 ∗ ln(Age) + 1.1434	0.1548
Parietal WM	− 0.044 ∗ ln(Age) + 1.1764	− 0.044 ∗ ln(Age) + 1.178	**0.0207**
Temporal WM	− 0.047 ∗ ln(Age) + 1.1969	− 0.046 ∗ ln(Age) + 1.1921	**0.0281**
Cerebellar WM	− 0.022 ∗ ln(Age) + 1.10272	− 0.01 ∗ ln(Age) + 1.0055	0.1283
Cingulum	− 0.044 ∗ ln(Age) + 1.1748	− 0.051 ∗ ln(Age) + 1.2236	1.3038
Corona Radiata	− 0.05 ∗ ln(Age) + 1.1911	− 0.051 ∗ ln(Age) + 1.2057	0.0864
Internal Capsule	− 0.036 ∗ ln(Age) + 1.1134	− 0.035 ∗ ln(Age) + 1.1096	**0.0091**
Optic Radiation	− 0.054 ∗ ln(Age) + 1.2089	− 0.051 ∗ ln(Age) + 1.2104	**0.0279**
Superior Longitudinal Fasiculus	− 0.04 ∗ ln(Age) + 1.1613	− 0.039 ∗ ln(Age) + 1.1442	0.1175

**Table 3:** Summary of extrapolated asymptotic myelin *g*-ratio index values for each brain region.

Region/tract	Left hemisphere asymptotic value	Right hemisphere asymptotic value
Frontal WM	0.77	0.78
Occipital WM	0.77	0.79
Parietal WM	0.77	0.77
Temporal WM	0.76	0.77
Cerebellar WM	0.9	0.84
Cingulum	0.77	0.75
Corona radiata	0.73	0.74
Internal capsule	0.78	0.79
Optic radiation	0.71	0.74
Superior longitudinal fasiculus	0.79	0.78

To examine the associations between the myelin *g-*ratio index, VF_M_, VF_F,_ ν_IC_, and ν_ISO_ more quantitatively, Pearson partial correlations were calculated (and converted to T statistics) between each white matter region and tract, while accounting for age. Partial correlations were calculated using *R*, version 2.3.1 (R Core Team, 2012). A summary of these results is provided in Table 4. Statistical significance was defined at p < 0.05 (uncorrected for multiple comparisons). Statistically significant partial correlations between the myelin *g-*ratio and VF_M_, VF_F,_ and ν_IC_ were found in all regions. Between myelin *g-*ratio index and ν_ISO_, statistically significant partial correlations were found in occipital white matter, left partial white matter, corona radiate, right internal capsule, and left optic radiation.

**Table 4:** Summary of partial associations (T-statistics) between myelin g-ratio index and VF_M_, VF_F_, ν_IC_, and ν_ISO_ over different brain white matter regions and pathways. Bold type denotes significant (p < 0.05, uncorrected) partial correlations, while taking into account ages.

Region/tract	VF_M_	ν_IC_	ν_ISO_	VF_F_
Body of corpus callosum	**− 10.260**	**− 2.608**	− 0.073	**− 4.686**
Genu of corpus callosum	**− 15.084**	**− 3.774**	− 1.277	**− 7.089**
Splenium of corpus callosum	**− 6.683**	**− 4.445**	− 1.194	**− 2.467**
Right white matter	**− 18.946**	**− 3.817**	− 0.432	**− 10.407**
Left white matter	**− 18.958**	**− 4.291**	− 0.706	**− 12.066**
Right frontal WM	**− 12.758**	**− 2.092**	− 0.303	**− 5.359**
Left frontal WM	**− 12.779**	**− 2.509**	0.108	**− 7.439**
Right occipital WM	**− 19.203**	**− 5.109**	**− 2.634**	**− 8.841**
Left occipital WM	**− 21.183**	**− 4.354**	**− 2.747**	**− 9.249**
Right parietal WM	**− 16.616**	**− 5.335**	− 1.428	**− 9.327**
Left parietal WM	**− 16.658**	**− 4.932**	**− 2.519**	**− 9.199**
Right temporal WM	**− 19.136**	**− 3.052**	− 1.104	**− 7.487**
Left temporal WM	**− 14.757**	**− 2.644**	− 1.072	**− 6.998**
Right cerebellar WM	**− 14.508**	**− 2.330**	− 1.273	**− 3.562**
Left cerebellar WM	**− 15.931**	**− 2.322**	− 1.735	**− 3.774**
Right cingulum	**− 12.796**	**− 3.245**	0.961	**− 6.187**
Left cingulum	**− 14.867**	**− 3.882**	− 1.955	**− 6.037**
Right corona radiata	**− 13.893**	**− 3.386**	**− 2.869**	**− 6.809**
Left corona radiata	**− 15.288**	**− 3.589**	**− 2.519**	**− 7.813**
Right internal capsule	**− 16.333**	**− 4.079**	**− 2.344**	**− 6.713**
Left internal capsule	**− 15.676**	**− 3.580**	− 1.603	**− 6.409**
Right optic radiation	**− 12.302**	**− 2.356**	− 0.291	**− 5.767**
Left optic radiation	**− 9.410**	**− 3.695**	**− 3.144**	**− 4.830**
Right superior longitudinal fasiculus	**− 16.249**	**− 2.858**	− 0.759	**− 7.059**
Left superior longitudinal fasiculus	**− 18.668**	**− 3.664**	− 1.536	**− 7.768**

Potential hemispheric developmental differences were also examined by fitting logarithmic models to myelin *g*-ratio index data from left and right hemisphere brain regions independently, as well as to the combined (left + right) hemisphere data. Residuals to these fits were then compared using an F-test with summary F-statistics shown in Table 2 revealing no significant hemispheric differences.

## Discussion

In this work, we have outlined a framework for calculating an index of the myelin *g-*ratio *in vivo* through the combination of myelin content measures derived from mcDESPOT, and neurite density information, estimated using NODDI. Though preliminary, our results in a small sample of children illustrate a logarithmically decreasing trajectory of *g*-ratio indices across infancy and early childhood that asymptotically approaches values aligned with theoretical predictions (Chomiak and Hu, 2009). These results suggest that this important parameter, which reflects the efficiency of white matter pathways and may, therefore, inform on brain network function, may be non-invasively investigated using MRI. This adds to a growing list of measures that reflect different, but complementary, aspects of white matter microstructure and development, including VF_M_, fractional anisotropy, radial and axial diffusivity, magnetization transfer metrics, to name a few (Alexander et al., 2011). As the first study of myelin *g*-ratio index changes across early neurodevelopment, we observe a rapid decrease in this measure during the first 600 days, which then slows and approaches a minimum value ranging between 0.71 in the optic radiations, and 0.9 in cerebellar white matter. The observed developmental trajectory of myelin *g*-ratio index broadly corresponds with the development and refinement of many early cognitive and behavioral functions (Casey et al., 2000, Casey et al., 2005, Johnson and Munakata, 2005, O'Muircheartaigh et al., 2014).

In addition to being consistent with histological measurements, the estimated values of the myelin *g*-ratio index in children using mcDESPOT-derived VF_M_ in conjunction with NODDI measures appears to be in agreement with the few studies that have presented similar MR-derived *g*-ratio maps(Campbell et al., 2014, Mohammadi et al., 2015, Stikov et al., 2015a). Though this is initially promising in regards to the proposed approach, it is important to note that considerable care must be taken in assigning the estimated myelin *g*-ratio index directly to this specific microstructure attribute. As the tissue model proposes, the quantities of the myelin volume fraction and fiber volume fraction are needed to estimate the *g*-ratio, assuming the *g-*ratio is constant within a voxel. However, estimation of the myelin or fiber volume fractions are likely to be limited by the respective MRI acquisition methods and quantitative techniques (Mohammadi et al., 2015). While previously described qMT methods (Campbell et al., 2014, Stikov et al., 2015a) have relied on linear relationships to estimate the myelin volume fraction from the bound pool fraction (F), in the current study, a proportionality coefficient relating VF_M_ to the myelin volume fraction was not used as such histological data is not currently available for mcDESPOT-derived VF_M_ measures. Such an assumption in the current approach may limit the interpretation of the presented myelin *g*-ratio index, as a mis-scaling of the VF_M_ from the myelin volume fraction could result in a coupling of the VF_F_ and the g-ratio index. Consequently, this could alter the statistical sensitivity of these measures, such as an implied g-ratio index change when there are only fiber density changes. However, determining the appropriate scaling coefficient that precisely relates F to the underlying myelin volume fraction may be challenging, as this proportionality coefficient is reported to vary widely throughout literature (Dula et al., 2010, Thiessen et al., 2013) and may be protocol dependent (Stikov et al., 2015a), and thus additionally limiting studies of the myelin *g*-ratio. Furthermore, this argument is equally true if such a proportionality coefficient exists between NODDI-derived VF_F_ and the true fiber volume density, though this was not previously accounted for in other studies (Campbell et al., 2014, Stikov et al., 2015a). It is thus critical for future studies to continue to examine and elucidate the underlying relationships between MRI-derived quantities and associated histological estimates, as well as explore the associations between MRI-derived quantities, such F and VF_M_.

The regional developmental trajectories (Fig. 5, Fig. 6, Fig. 7) highlight myelination and increases in myelin thickness occur at different stages of development for specific brain regions. This regional variability is consistent with previous histological (Barkovich et al., 1988), conventional and quantitative MRI studies (Dietrich et al., 1988, Evans and Brain Development Cooperative Group, 2006, Giedd et al., 1999, Giedd et al., 1996, Kulikova et al., 2014, Prastawa et al., 2010, Westlye et al., 2009). Interestingly, the trajectory of cerebellar white matter was distinct. Although cerebellar white matter followed a similar logarithmic shape, it developed at a slower rate and asymptotically approached higher myelin *g*-ratio index values than other brain regions. The cerebellum is one of the earliest brain regions to develop, with the most rapid development beginning prenatally (Dobbing and Sands, 1973). The slower rate of development may, therefore, be due to the lack of imaging data prior to 3 months of age. Higher *g*-ratio index values later in development, however, may result from cerebellar white matter being composed of larger axons (Necchi et al., 2008), which have higher *g*-ratio values and may be indicative of thinner myelin sheaths (Berthold et al., 1983, Gillespie and Stein, 1983).

Myelin is a critical component of CNS tissue, and the maturation of the myelinated white matter is a central process of early brain development. Myelinated axons play a key role ensuring synchronized transmission and information integration across neural systems, and myelinated axons underlie normative brain functioning (Fields, 2008, Fields, 2005). A particularly interesting observation is the relationships noted between measured myelin *g****-***ratio index values and the other quantitative measurements derived from mcDESPOT and NODDI (Table 4). The presented results, which show partial correlations between VF_M_, VF_F_, v_IC_, and ν_ISO_ while taking into account of age, highlight the strong dependence of the myelin *g*-ratio index on VF_M_, VF_F_, and v_IC_. These relationships are not surprising as each of these parameters is highly influenced by changes to the myelinated white matter and contributes to the estimation of the myelin *g*-ratio index. It is possible that such correlations exist in tissue and hence we would expect such correlations between these MRI metrics. However, it is equally possible that the correlations between these *in vivo* parameters exist due to flaws or invalid assumptions of the framework. Hence, it is important that the comparisons of the myelin *g*-ratio index and other MRI parameters be substantiated with histological studies as well as in larger cohorts of similarly aged individuals to ensure these measures are indeed complimentary. Of the various available measures of white matter integrity, myelin thickness may offer unique insight into axonal and white matter pathway conduction velocity and efficiency (Melbourne et al., 2014). Myelin *g*-ratio indices, therefore, may provide important new insight into brain network function and afford improved understanding and prediction of cognitive performance and outcome. For example, an increased myelin *g*-ratio index may reflect decreased myelin thickness, such as is observed in early infancy (Fig. 4, Fig. 5, Fig. 6).

As myelination proceeds over the first two years, we observe a rapid change in the *g*-ratio index that reflects the complexity of early brain development. As these *g*-ratio indices may be sensitive to myelin sheath thickness, *g*-ratio indices above expected values, could, therefore, be indicative of damage to the myelin sheath, such as in hypomyelinating or demyelinating disorders. Consequently, mapping myelin *g*-ratio index may provide additional information in hypomyelinating leukodystrophies, or multiple sclerosis. In contrast, decreased myelin *g*-ratio indices may suggest an over or hyper-myelination that could result from inefficient wrapping of the myelin sheath by oligodendrocytes or genetic mutations that result in the overproduction of myelin (Paus, 2010). As myelination is tightly regulated by both genetics and neuron activity (Fields, 2005), both increased and decreased myelin sheath thickness can affect axonal conduction velocity and efficiency, resulting in disrupted brain messaging and abnormal brain functioning (R. S. Smith and Koles, 1970, Waxman, 1980). Changes to the myelin *g*-ratio index may be indicative of other neurodevelopmental mechanisms, such as increases of axonal conduction velocity (Purves et al., 2001, Waxman, 1980). For instance, increases in the size of intra-axonal fibers (*i.e.* fiber diameter increases), will result in an increase in the myelin *g*-ratio index, though only if fiber increases are not balanced with myelination. Conduction velocity, on the other hand, is directly related to the myelin *g*-ratio (Johansen-Berg and Behrens, 2013), and thus estimates of the myelin *g-*ratio may be informative to structural and functional brain connectivity studies (Mohammadi et al., 2015), as well as inform on the overall efficiency of information transfer in specific white matter tracts and networks. Interpretation of the myelin *g-*ratio index within regions of gray matter, and particularly unmyelinated brain regions are unclear. While recent advances in MRI scanner hardware and image resolution could make measures of the myelin g-ratio index applicable to studies of the cortical myeloarchitecture (Deoni et al., 2015, Glasser et al., 2014, McNab et al., 2013), the sensitivity of NODDI and mcDESPOT imaging techniques to unmyelinated brain regions should also be examined, as thse may provide improved insight into the significance of the myelin *g*-ratio index in gray matter. Moreover, as white matter abnormalities and atypicalities of the myelin *g*-ratio are thought to underlie neurodevelopmental and psychiatric disorders (Fields, 2008, Nagy et al., 2004), *in vivo* measurement of myelin *g*-ratio index may offer a new perspective to understanding fundamental white matter alterations.

In addition, estimation of the myelin *g*-ratio index may also provide new insights into typical brain development. Prior cross-sectional and longitudinal studies have sought to develop quantitative models of white matter development (Dean et al., 2014b, Lebel and Beaulieu, 2011). However, the developmental trajectory of the myelin *g*-ratio is unknown. While we fit logarithmic models to the data herein, these models begin to deviate in the older ages (*i.e.*, beyond 1500 days in Fig. 5, Fig. 6, Fig. 7). Models that more accurately reflect and characterize the evolution of *g*-ratio index with age may be realized by both examining additional biological growth models (Dean et al., 2014a), and extending the sample in age and sampling density. Moreover, longitudinal study designs that utilize mixed-effects modeling approaches (Dean et al., 2014b, Lebel and Beaulieu, 2011, Travers et al., 2014) may be informative about the individual variation of the myelin *g*-ratio index across early childhood development.

Potential applications are not limited to the study of early neurodevelopment but include the study of neurodevelopmental and psychiatric disorders, typical and atypical aging, plasticity, and structure–function relationships. For example, it is likely that these age-related changes in white matter maturation and myelination reflect changes in brain activity. Studies combining resting-state fMRI (Alcauter et al., 2015) or electroencephalography (EEG) measurements (Dubois et al., 2008, Horowitz et al., 2014, Meyer-Lindenberg, 1996) with maps of the myelin *g*-ratio index could be informative of the dramatic changes in inter- and intra-hemispheric connectivity and underlying functioning of the neural circuits during early brain development. Furthermore, while the current study did not investigate differences between males and females due to the small sample size and the disproportionate number of males and females, male–female differences in the brain have more recently hypothesized to arise from differences in the myelin *g*-ratio (Paus and Toro, 2009, Pesaresi et al., 2015). We have previously reported regionally dependent differences in myelination trajectories across this period of early neurodevelopment (Dean et al., 2014b, Deoni et al., 2012), and therefore we anticipate myelin *g*-ratio index measurements to be sensitive to *g*-ratio differences between males and females. Hence, studies utilizing combined mcDESPOT and NODDI acquisition techniques to estimate the myelin g-ratio index may provide a flexible framework to the study of white matter.

Despite the promise of *in vivo* myelin *g*-ratio imaging, potential limitations exist with the present study. First, the limited sample size challenges our ability to investigate hemispheric and sex differences. Larger cross-sectional and longitudinal studies examining the changes of the myelin *g*-ratio index throughout development are needed to address such questions. Second, we acknowledge the lack of histological validation of the utilized tissue model. Thus, there may be concerns regarding the accuracy and reproducibility of MRI-based *g*-ratio index measures. The tissue model used here relies on the assumption that a single value is capable of characterizing the myelin *g-*ratio of all the axons within a voxel (De Santis et al., 2015). Such assumptions may be overly simplistic as studies have more recently shown the axon caliber to vary within a voxel (Assaf et al., 2008, De Santis et al., 2015, West et al., 2015; H. Zhang et al., 2011). Computing an overall distribution of the myelin *g*-ratio index at each voxel may be more appropriate to characterize the various fiber populations that reside in a voxel, however, such approaches likely suffer from long acquisitions and high computing demands, though such implementations are of interest for future work. Though the *g*-ratio index values calculated here agree well with prior theoretical models (Chomiak and Hu, 2009, Rushton, 1951), and histologically-derived measures (Berthold et al., 1983, Chomiak and Hu, 2009, Goldman and Albus, 1968) of the myelin *g*-ratio, the majority of this past work has involved animal and adult samples and, thus, may differ from the pediatric values reported here. We attempted to address this by extrapolating the pediatric trajectories (Table 3). Stikov et al., 2015a, Stikov et al., 2015b has additionally showed histological measurements of the myelin g-ratio in the macaque corpus callosum to be consistent with aggregate MRI g-ratio estimates (Stikov et al., 2015b, Stikov et al., 2015a), giving promise to the underlying tissue model. Nevertheless, such studies have thus far been limited and further investigation of MRI based g-ratio indices within animals and adults are needed for more thorough comparative analysis, while the dependence of *g*-ratio index estimates on multiple fiber populations should additionally be investigated.

In addition to the concern about the actual tissue model, there is also uncertainty regarding the specificity and accuracy of the myelin content and neurite density information. In particular, mcDESPOT values are known to be elevated compared to conventional multi-echo spin-echo measures (J. Zhang et al., 2015) and have yet to be histologically validated in humans (Lankford and Does, 2013). We have noted strong qualitative agreement between histology and mcDESPOT in a Shaking Pup model of dysmyelination (Hurley et al., 2010), while mcDESPOT VF_M_ maps of early neurodevelopment qualitatively agree with known spatial–temporal profile of histological myelin measurements (Flechsig, 1901) and have further been shown to reflect clinical impairment in white matter pathologies, such as multiple sclerosis (Kitzler et al., 2012, Kolind et al., 2012), amyotrophic lateral sclerosis (Kolind et al., 2013), and epilepsy (Spader et al., 2013). Such studies give confidence that mcDESPOT is strongly sensitive, if not specific, to myelin content. Moreover, despite the lack of direct histological validation of NODDI, neurite density measures derived from diffusion imaging data has been shown to be comparable to histological measures (Jespersen et al., 2010). Future histological studies are thus critical for quantitatively evaluating these novel and informative microstructural imaging techniques.

Finally, contrary to the mcDESPOT acquisition, the NODDI protocol has not been tuned for different ages in relation to different brain sizes and diffusion properties. While the quality of the NODDI data was not observed to be impaired by the choice of parameters used in the current study, it may have been possible to reduce the NODDI acquisition if such an optimized protocol had been developed (Kunz et al., 2014). Moreover, we adhered to using the default adult diffusivities provided in the NODDI MATLAB toolbox (H. Zhang et al., 2012) for the estimation of the NODDI model quantities. Such an assumption may be valid in the developing brain provided that the central microstructural differences between a child and adult are developmental differences of myelination and fiber density, which may lead to considerable differences in diffusion metrics(Hüppi and Dubois, 2006, Kunz et al., 2014, Lebel et al., 2012). Nevertheless, the development of efficient NODDI acquisition protocols and examining the effects of the underlying NODDI model assumptions, such as the default diffusivities, on parameter estimates are critically important for utilizing the NODDI technique, as well as the presented *g*-ratio index mapping approach, in studies of early neurodevelopment.

Integrating parametric imaging information from mcDESPOT and NODDI represents a unique and non-invasive technique for quantifying an index of the myelin *g*-ratio *in vivo*. For the first time, we have presented developmental trajectories of this myelin *g*-ratio index during early childhood. This presented work provides an important step for understanding the developmental patterns of white matter microstructure and will facilitate future studies examining the role of the myelin *g*-ratio throughout brain maturation.

## Figures and Tables

**Supplementary Fig. 1:**
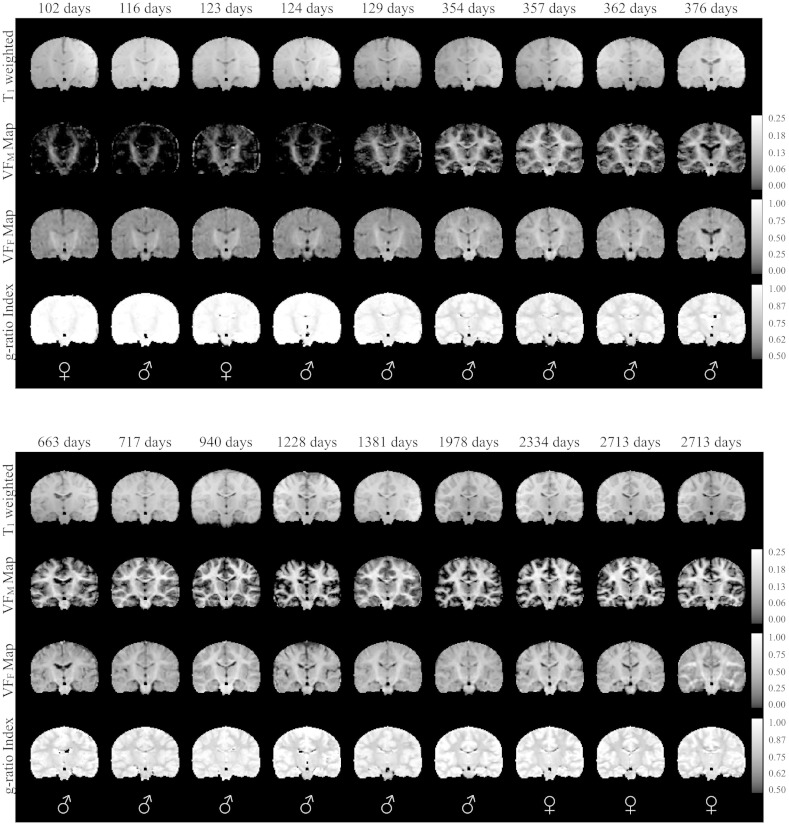
**:** Coronal T_1_-weighted and quantitative VF_M_, VF_F_, and myelin *g*-ratio index maps for each of the typically developing subjects. Images are shown in radiological convention (viewing left = anatomical right). The sex of each infant is denoted in the bottom panel. Note: while *g-*ratio in gray matter has limited interpretation, these areas have not been masked in the *g*-ratio index maps so that changes in myelination can be better appreciated.

**Supplementary Fig. 2:**
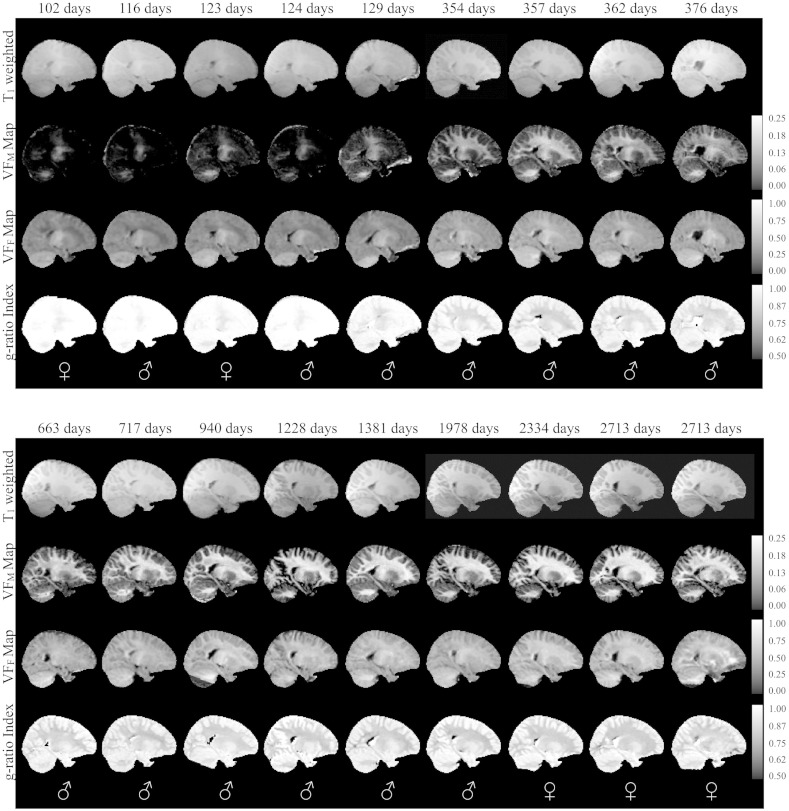
**:** Sagittal T_1_-weighted and quantitative VF_M_, VF_F_, and myelin *g*-ratio index maps for each of the typically developing subjects. Images are shown in radiological convention (viewing left = anatomical right). The sex of each infant is denoted in the bottom panel. Note: while *g-*ratio in gray matter has limited interpretation, these areas have not been masked in the *g*-ratio index maps so that changes in myelination can be better appreciated.
